# Supplementary material for: “Living an Obstacle Course”: A Qualitative Study Examining the Experiences of Caregivers of Children with Rett Syndrome
Source: Int J Environ Res Public Health. 2018 Dec 25;16(1):41. doi: 10.3390/ijerph16010041 (PMC6338949; doi:10.3390/ijerph16010041)
Supplement: Supplementary file 1 [file ijerph-16-00041-s001.pdf]

Supplementary Material

"Living an Obstacle Course": A Qualitative Study Examining the Experiences of Caregivers of Children with Rett Syndrome

Domingo Palacios-Ceña, Pilar Famoso-Pérez, Jaime Salom-Moreno, Pilar Carrasco-Garrido, Jorge Pérez-Corrales, Paula Paras-Bravo and Javier Güeita-Rodriguez

Table S1. Demographic and clinical features of the participants.

Parent number	Age (years)	Sex of parent	Number of children	Age (years) of child with RTT	Sex of child with RTT	Age (months) when first symptoms were identified	Person who identified first symptoms	Age (years/months) at diagnosis	Clinical diagnosis *	Genetic diagnosis **
P1	63	Female	2	4	Female	12	Mother	2y		MECP2
P2	33	Female	2	4	Female	12	Mother	2y		MECP2
P3	35	Male	2	4	Female	12	Mother	2y		MECP2
P4	45	Female	1	9	Female	15	Mother	2y	YES	Without a genetic dx
P5	48	Male	1	9	Female	15	Mother	2y	YES	Without a genetic dx
P6	38	Female	1	6	Female	12	Mother and pediatrician	3y 10m		MECP2
P7	43	Male	1	6	Female	12	Mother and pediatrician	3y 10m		MECP2
P8	45	Female	2	11	Male	3	Mother and physiatrist	3y		DCKL5
P9	46	Male	2	11	Male	3	Mother and physiatrist	3y		DCKL5
P10	42	Female	2	5	Male	9	Mother	2y 6m		DCKL5
P11	46	Female	3	11	Male	At birth (microcephaly)	Doctor	10y		FOXG1
P12	49	Male	3	11	Male	At birth (microcephaly)	Doctor	10y		FOXG1

P13	37	Female	2	12	Male	4	Nurse	7y		MECP2
P14	42	Male	2	12	Male	4	Nurse	7y		MECP2
P15	46	Female	1	14	Male	At birth (microcephaly)	Doctor	8y		FOXG1
P16	48	Male	1	14	Male	At birth (microcephaly)	Doctor	8y		FOXG1
P17	41	Male	2	5	Female	10	Father	1y 8m		MECP2
P18	36	Female	2	5	Female	10	Father	1y 8m		MECP2
P19	39	Male	2	12	Female	Prenatal detection (ultrasound)	Doctor	3y		MECP2
P20	36	Female	2	12	Female	Prenatal detection (ultrasound)	Doctor	3y		MECP2
P21	55	Female	2	16	Female	14	--	4y	YES	Without a genetic dx
P22	63	Female	2	31	Female	At birth (epilepsy)	Doctor	4y	YES	Without a genetic dx
P23	67	Male	2	31	Female	At birth (epilepsy)	Doctor	4y	YES	Without a genetic dx
P24	43	Male	3	17	Female	10	--	2y 6m		MECP2
P25	67	Male	2	38	Female	8	Grandmother	16y clinical dx 22y genetic dx	YES	MECP2
P26	65	Female	2	38	Female	8	Grandmother	16y clinical dx 22y genetic dx	YES	MECP2
P27	53	Male	2	19	Female	12	--	1y 6m		MECP2
P28	46	Male	1	12	Female	12	--	1y 6m		MECP2
P29	35	Female	2	7	Female	12	Mother	1y 6m		MECP2
P30	30	Female	2	7	Female	15	Mother	1y 6m		MECP2
P31	25	Female	1	3	Female	12	Mother	1y 8m		MECP2

* Diagnoses based identify signs and symptoms, not genetic diagnosis confirmation; ** Diagnoses based and confirmed at genetic determinations; dx= diagnosis; y= years; m= months.

Table S2. Narratives of caregivers of children with Rett syndrome.

Themes	Subthemes	Caregivers' narratives
Looking for answers	The first symptoms	**Perception of changes:** *"…suddenly one day you detect problems… and you feel that it is beyond your understanding." (P2, 33 years old). "You begin to see things, difficulty moving, swallowing, and little by little you realize that you only see difficulties", (P8, 45 y.o) "It was tough realizing that one day you only saw problems, your daughter was unable to do many things…" a P8, 45 y.o)*
		**Presentation of symptoms:** *"You didn't know when they were real problems or when they were things in your head, you know, being overprotective and all, because it's the first time… Also, they were never the same things, one day, problems for swallowing might arise, another day, there were difficulties regarding movement, they were never the same… there wasn't a clear pattern…" (P5, 48 y.o)." "Some days the symptoms get worse and others they hide and almost don't appear, you don't know what to think." (P10, 42 y.o) "We have learnt that we can't have many expectations. The illness is there and if one day it doesn't give you any difficulties, it doesn't mean that it's gone, it's still there" (P10, 42 y.o)*
		**Difficulties recognizing symptoms**: *"The first thing was verbalizing it… it was a big step, because you are seeing it, but it's as if you don't want to see it." (P15, 46 y.o). "Everything happens in front of your eyes, you don't believe until it is too late"(P2, 33 y.o). "You always believe that, as a mother, you will realize immediately if your daughter has anything… I had to learn that it isn't so, not with this disorder"(P7, 43 y.o) "I couldn't believe it, I had been taking care of her every day since she was born, I thought that with time, something that I had seen would change or improve, but it wasn't so …"(P6, 38 y.o.)*
		**Who identified the symptoms?:** *"… My child doesn't do what that boy does … At 15 months my daughter constructed four-word sentences… She began to lose them … At the age of 2 she didn't want to walk." (P29, 35 y.o) "I always tried to do tests to confirm my fears, I saw how he didn't do things like other children, I was the first [to detect it]" (P6, 38 y.o) "I didn't want to tell my husband, what if I was wrong and I got everyone worked up for nothing? But I couldn't avoid feeling it inside. Something was wrong and I couldn't stop it." P6, 38 y.o)*
		**Nursery school and early detection***: "At three months I already took her to nursery… And, of course the girls of her age, they had to change the class she was in, they didn't want to be with my daughter… Also, my daughter learnt at a slower pace than the other children." (P18, 36 y.o) "The key moment is when you take your child to spend time with other children in the nursery school, that's the best test" (P9, 46 y.o) "You hear so many things, other caregivers tell you that in early years in nursery school you see many things… When the teacher called me from the nursery schools, and started telling me… my heart sank" (P13, 37 y.o)*

The need for a diagnosis	**The need for a diagnostic confirmation**: *"We searched for someone to tell us what was happening to our daughter, what it was called, a name, something to justify everything that we were going through." (P21, 55 y.o). "Nobody knows anything, and, sometimes you think that they don't want to risk saying the name of the problem. But we want and need the name of what is happening with our daughter" (P13, 37 y.o)*<br><br>**The search for information:** *"We had no idea that we were going to ask an army of specialists… Nobody knew anything… There were no certainties or anything… Anything was possible… a syndrome… but nothing concrete… The hardest thing was finding a diagnosis." (P12, 49 y.o) "The information is not an option, it's a need, it helps us to know what we have on our hands and what we can expect, it helps us be prepared." (P22, 67 y.o)*<br><br>**The importance of a genetic diagnostic**: *"From the beginning we asked for a genetic test, however we always got a negative response. They said that there were many things to rule out and that many other tests were necessary beforehand, before opting for the genetic diagnosis… Ruling out other pathologies delayed everything, made us feel more anxious. If it was clear to us, why wasn't it clear to the doctors? The genetic testing was key for initiating the treatment." (P30, 30 y.o). "It's like a final proof, if the genetic diagnosis is positive, it opens the path to continuing a more focused treatment, and, above all, knowing what my daughter has. If you don't have it, everything is just speculations, and nobody does anything." (P21, 55 y.o)*<br><br>**The child's sex**: *"What hurt me the most after knowing that my son is an atypical Rett Syndrome was knowing that many doctors do not prescribe genetic Rett tests in males because they think that it doesn't occur in males…" (P6, 38 y.o) "The first thing the doctors discard is Rett because he is a boy, as he doesn't fulfill what is statistically expected, they discard it almost without even performing a test. And although you tell them that you see the Rett symptoms, one by one, they don't take any notice of you." (P6, 38 y.o)*<br><br>**The search for a diagnosis**: *"What illness? At each center we went to, we were given a different name. First Cornelia de Lange. When she suffered epileptic seizures… Then West Syndrome… We stopped seeking a name, because it was absurd, we just wanted to help her… We ended up putting her in a special school… In the autism classroom, despite not knowing exactly what she had… We couldn't delay it anymore because of the diagnosis, we had to do something at the time. (P13, 37 y.o). "The diagnosis is like the search for the Grail or like an adventure… you never know when to stop, everything is confusion, and nothing is clear, everyone has their own perspective and everyone has good arguments, you don't know what to do or who to listen to" (P27, 53 y.o)*<br><br>**A multitude of diagnostic tests**: *"Thousands of tests and they didn't show anything. We suffered for our daughter, some were painful… The neurologist of the Social Security had a suspicion and decided to request further tests, very strange tests … the tests took… two years. All that lost time, as the results came back negative…" (P4, 45 y.o) "We know that tests are necessary, however, a*

		time comes when you ask yourself; What for? I know that the tests are necessary, but there comes a time when you ask yourself, what for? I know I am not going to cure her, why do more things?" (P13, 37 y.o)
Managing day to day life	Applying treatments	**The medical check-ups**: *"He has five doctors, five checkups. And when I go I stay all day … a Neurologist, a Gastroenterologist, a Pneumologist, a Physiatrist, a Cardiologist… All on the same day … I prefer it like that as I don't miss so many days at work. Also, for me it's best as I live very far away." (P5, 48 y.o). "Sometimes you end up laughing about it with the family, laughing out of despair, you don't know how to do it, it seems like the agenda of a politician or a rock star, he has so many appointments, you don't know how to do it" (P11, 46 y.o) "Everything is centered around the health world. I have started to get to know as if it were my job, because of so many hospital visits." (P20, 36 y.o)*<br><br>**The administration of medication**: *"A big problem is how antiepileptic medicine is administrated, they have to ask us how to give them to her. [We have to administer it] Without yoghurt or food that she can spit out. In the case of my daughter we have to tilt her backwards and put the drop on the tongue, as, otherwise, it gets lost." (P18, 36 y.o).*<br><br>**Consequences of not being able to administer the medication**: *"If you are unable to give her the pill, she may end up having a seizure, then you take her to the doctor and they increase the medication." (P18, 36 y.o). "Giving oral medication is a struggle… Not managing for him to take medicine through the mouth means that they may place a tube through the nose to the stomach, in order to administer the medication." (P27, 53 y.o)*<br><br>**The need for non-pharmacological therapies**: *"For my daughter, going to therapy is the same as the extracurricular activities at school. The difference is that she needs these therapies to live. She needs something more besides medication in order to face this illness." (P2, 33 y.o) "There comes a time when you realize that it is necessary to include more things, more experiences, not just medication or pills." (P14, 42 y.o)*<br><br>**Physiotherapy as a fundamental pillar**: *"All they have is physiotherapy, we have always had to fight for them to give it to her. One or two weeks, then you are discharged and out." (P31, 25 y.o). "I have learnt to consider it as the fundamental pillar in the care of my daughter… if you ask me what I need, I will respond that what I could never go without is physiotherapy." (P20, 36 y.o)*<br><br>**Other therapies**: *"We try the things that she likes, one day we go to the pool, we go to the physiotherapist, music therapy and we rest. We may even have two days of rest." (P4, 45 y.o). "We are programming more therapies, so that she can adapt to other things, we*

	believe that a certain point in time will come when, the more variety of things she has, something will work, or everything together will make her better." (P4, 45 y.o)
Learning to care	**Learning to care**: *"We had no idea whatsoever. It is like learning everything over again… You don't even know how to take care of your own daughter… Sometimes you get it right, others you don't… What works one day, another day no longer works…" (P19, 39 y.o) "Everybody tells you things in a simple manner so that you can understand and apply it, but the nurse tells you one thing, the doctor tells you another thing, the therapist says something else, a lot of little things in the end are many things!! (P30, 30 y.o)"It's hard to have to learn things that you never imagined existed, pressure sores, orthoses… I almost didn't even know what they meant" (P26, 65 y.o)*<br><br>**Parental concerns**: *"He had a very severe malnutrition… He ate three spoonfuls and then he didn't eat anymore… They put him on gastric tube feeding… but finally they did a gastric button, and since then he has gotten strong… He is well fed"(P5, 48 y.o). "The worst thing is worrying that whatever I do, I can't resolve some of the problems that arise from day to day…" (P25, 67 y.o) "Sometimes I feel that I don't fulfill my role as a father… my mission is to protect my daughter, and, sometimes, there are so many symptoms that I don't know if when I do something I may be worsening another" (P31, 25 y.o)*<br><br>**Learning to prepare lunch**: *"An everyday activity such as eating turns into a challenge and an art, the mix of nutritional foods, rich in everything such as vitamins, minerals, proteins, etc. As well as how to cook and prepare meals so that these can be easily digested…" (P26, 65 y.o) "To eat is to eat, right? Actually, it isn't, it's difficult even for her to swallow food, sometimes you have to take a masters degree to prepare breakfast" (P12, 49 y.o) "I never thought that it would be so complicated, it's eating, that's all, I thought Rett could give other big and vital problems, but it places obstacles on all the small things: moving, breathing, eating, little by little, everything, up to feeding themselves" (P19, 39 y.o)*<br><br>**Bladder and bowel elimination**: *"When she is constipated she is very agitated, the fecalomas that she produces are very big and this makes her intestines become dilated, which hurts her, and the more dilated they are, the more sensitive it is, therefore she gets a tremendous dry ball. She really suffers! We have had to learn to mix laxatives, enemas, get them out with our finger, everything." (P15, 46 y.o). "Sometimes she suffers when she realizes that she is peeing on herself and the clothes are getting soiled, I try to calm her down, clean her and dry her as soon as possible." (P5, 48 y.o)*<br><br>**Sleeping difficulties**: *"The worst thing of all, is not sleeping. Sometimes because she has pains, she is agitated, and others because she is exhausted, you don't know how to manage it… But the worst thing is the accumulated tiredness and the feeling of getting burned out*

	and the fear of making a mistake with something and provoking further pain." (P29, 35 y.o). "Often you are exhausted, not just because you are unable to sleep, but because you can't get her to sleep. It's not nonsense. It also affects her, and I can't imagine how tired she can be." (P7, 43 y.o)<br><br>**Epileptic seizures**: *"I was with the neighbor, the seizure was so big that he almost ended up dead. The ambulance came and they resuscitated him, because he was clinically dead … Now, every time I go to the toilet I am calling him constantly, because when I talk to him he laughs and that way I hear him. Maybe I go over the top and am too vigilant, but we know what the other alternative is and I am not going to risk it." (P8, 45 y.o). "The epileptic seizures are very tough, I don't know what to fear the most, the uncertainty or the epileptic seizures. Whether or not I will be there to help… My heart sinks just thinking about it." (P8, 45 y.o).*<br><br>**Epileptic seizures and menstruation**: *"They called me from the school, they had to take my daughter to the hospital because she had suffered another seizure … The career tells me she was very unsettled and nervous and she became more and more agitated until she had the seizure… I looked at her clothes and she had gotten her period. From that day on, the checkups are very precise, we don't let anything pass us by. My daughter has a very painful period/premenstrual syndrome." (P20, 36 y.o). "Now this, at all times, keeping a close watch on the period, when it will come, when it doesn't come, the frequency, and those things, it can't happen again and it can be an alarm signal that helps me to prevent it" (P20, 36 y.o).*<br><br>**Stereotypical movements**: *"Also, she stuck her hand far back in her mouth, this kind of movement that I can't do, it makes me queasy, it is a very typical stereotypical movement, and the truth is we had a hard time with it" (P17, 41 y.o). "Sometimes I have to explain to family members or visitors about these stereotypical movements, but it is hard to understand" (P17, 41 y.o) "This continues to be what impacts me the most. I can see it everyday, become aware that it is part of the syndrome, but I continues to affect me" (P12, 49 y.o)*<br><br>**Uncontrolled movements**: *"Awful, she hit her head against the wall… the screams… argh! Totally aggressive! She had self-injurious behavior."P18, 36 y.o), "I observe that all the furniture in the living room is covered with protective foam and stuck on with masking tape, to avoid any type of edge or ridge that could cause an injury."* (Researcher notes) *"I am really scared of the movements that I can't predict, in case she hurts herself." (P19, 39 y.o)*
Money matters	**Impact on economic resources**: *"…on top of everything, the financial effort we have to make, they have us cornered in all around, the financial effort that I am going to have to make, to try and get my daughter to have the best possible options… A single family has to have lots and lots of money to be able to cope with everything that this illness entails" (P3, 35 y.o), "Beforehand, my husband and I worked. But now we have spent everything we had and I no longer work… I have to take care of my daughter, we cannot hire anyone to take care of her." (P6, 38 y.o). "When you go to a specialist, you can't avoid translating everything they say to how much money it will*

cost and how I am going to manage it or to how many shifts I am going to have to work to achieve it, it's horrible, it sounds awful, but that's the way it is."P17, 41 y.o)

Treatments that are not covered: *"It comes out of our pocket and that of the grandparents … because she needs a physiotherapist and a speech therapist every day. All this treatment is individual and private. Up to four professionals work with her." (P2, 33 y.o). "We noticed it especially regarding wanting a physiotherapist every day… We did it the first months, then the money ran out and we had to reduce it to three times a week, then one day… until now"(P27, 53 y.o), "There are therapies that you would like to try, horses, dolphins, animals, etc. But directly unfortunately it isn't worth it, one day of those therapies means 3 days of other therapies."(P29, 35 y.o)*

The cost of health resources: *"It is stratospheric… All the orthopedic aids, it is insulting what they cost… They take advantage of the imperative and unavoidable needs of people attempting to have a half decent life…" (P21, 55 y.o). "Anything costs a lot, a splint, a prosthesis, a crutch. Goodness me, everything is expensive" (P26, 65 y.o) "The worse thing is that any help that they propose costs money, anything that they tell you costs money… and a lot of it" (P21, 55 y.o)*

Adapting the home: *"I need a wheelchair, but it doesn't fit through the kitchen or the bathroom, I have carry out reforms. The bedroom is another story, sometimes I can't have more things because there is no space…" (P11, 46 y.o). "I never imagined I would have to change the whole house. These are subtle things which however mark a difference between giving my daughter quality or not" (P19, 39 y.o) "There was a before and after, the house, just like everything else, not only did we change the family, but also our home" (P24, 43 y.o)*

Unequal opportunities: *"I need to know what is wrong with my child. Great, you need to spend 400 euros on the genetic diagnosis. Who can pay that amount all of a sudden, without planning for it? If you don't have the money they don't give you any other option besides accepting a non-conclusive diagnosis." (P12, 49 y.o) "It's not true that we all receive the same treatment and that our daughter is treated the same, you only have to see the amount of time that passes for the diagnosis among families…"(P10, 42 y.o) "It's clear, whoever has money and resources can do more for their daughter, that's obvious." (P18, 36 y.o)*

Help via research projects: *"And we know the diagnosis because the doctor was given a specific grant to study these cases" (P13, 37 y.o), "At the Association there was an agreement with San Juan de Dios [Hospital Foundation] and a grant was requested to the Health Research Fund, they gave us some money so that they could begin the genetic testing." (P21, 55 y.o). "We don't like it, but research may be a solution for our daughter" (P17, 42 y.o) "We don't know what will happen or whether the treatment or research will work, but at least it's a possibility, better then nothing," P14, 42 y.o)*

Seeking solutions and solidarity amongst families: *"At a market we saw this guy, selling walkers for disabled people at the markets" It was very dirty, but I said, I want this walker for my son, I cleaned it, we put wheels on it and there it is… Also, it belonged*

	to a girl, who had passed away." (P12, 49 y.o). "We have found more support among families than from the public or other entities" (p7, 41 y.o) "Above all, we received help from other families and caregivers with the same problem, people who had gone through what we had gone through." (P15, 46 y.o)
